# Organizational attributes that contribute to the learning & improvement capabilities of healthcare organizations: a scoping review

**DOI:** 10.1186/s12913-023-09562-w

**Published:** 2023-06-07

**Authors:** Kees de Kok, Wilma van der Scheer, Corry Ketelaars, Ian Leistikow

**Affiliations:** 1 Dutch Health and Youth Care Inspectorate (IGJ), Stadsplateau 1, 3521 AZ Utrecht, The Netherlands; 2 Health Care Governance, Erasmus School of Health Policy & Management, Erasmus University, Burgemeester Oudlaan 50, Rotterdam, The Netherlands

**Keywords:** Organizational attributes, Learning capabilities, Improvement capabilities

## Abstract

**Background:**

This study aims to explore and identify the organizational attributes that contribute to learning and improvement capabilities (L&IC) in healthcare organizations. The authors define learning as a structured update of system properties based on new information, and improvement as a closer correspondence between actual and desired standards. They highlight the importance of learning and improvement capabilities in maintaining high-quality care, and emphasize the need for empirical research on organizational attributes that contribute to these capabilities. The study has implications for healthcare organizations, professionals, and regulators in understanding how to assess and enhance learning and improvement capabilities.

**Methods:**

A systematic search of peer-reviewed articles published between January 2010 and April 2020 was carried out in the PubMed, Embase, CINAHL, and APA PsycINFO databases. Two reviewers independently screened the titles and abstracts and conducted a full-text review of potentially relevant articles, eventually adding five more studies identified through reference scanning. Finally, a total of 32 articles were included in this review. We extracted the data about organizational attributes that contribute to learning and improvement, categorized them and grouped the findings step-by-step into higher, more general-level categories using an interpretive approach until categories emerged that were sufficiently different from each other while also being internally consistent. This synthesis has been discussed by the authors.

**Results:**

We identified five attributes that contribute to the L&IC of healthcare organizations: perceived leadership commitment, open culture, room for team development, initiating and monitoring change, and strategic client focus, each consisting of multiple facilitating aspects. We also found some hindering aspects.

**Conclusions:**

We have identified five attributes that contribute to L&IC, mainly related to organizational software elements. Only a few are identified as organizational hardware elements. The use of qualitative methods seems most appropriate to understand or assess these organizational attributes. We feel it is also important for healthcare organisations to look more closely at how clients can be involved in L&IC.

**Trial registration:**

Not applicable.

**Supplementary Information:**

The online version contains supplementary material available at 10.1186/s12913-023-09562-w.

## Introduction

Citizens count on good quality of care when they need it, but in practice, there can be large differences between healthcare organisations in regard to quality, despite quality management systems, procedures for accreditation, regulation and the intrinsic motivation and efforts of healthcare workers to provide the best quality of care [1–6]. According to contemporary insights, quality of care is more of a dynamic concept than a static concept; it is not only relationally but also organizationally determined [7–9]. In addition, the context can also change and lead to new insights about quality of care. For instance, the recent COVID-19 pandemic demonstrates the importance of healthcare organisations having the ability to learn in rapidly changing circumstances and adapt and improve the quality of care accordingly [9, 10]. The simultaneous occurrence of significant differences in quality, the awareness that quality of care is strongly relationally and organizationally determined, and the intrinsic motivation of healthcare professionals stresses the importance of the learning and improvement processes that take place within healthcare organizations.

In this study we aim to examine the concepts of learning, improvement, and learning organization and identify the organizational attributes that contribute to learning [11] and improvement [12] capabilities (L&IC) in healthcare organizations.

In line with Barron, we define learning as a structured update of system properties based on the processing of new information [13]. This new information can be obtained both formally and informally.

Improvement is a phenomenon that is difficult to define in a universal way, as it is socially constructed and dependent on various circumstances. For this study we draw on the working definition of improving public services: improvement occurs if there is a closer correspondence between perceptions of actual and desired standards [14]. In our view, improvement is a dynamic concept, in which both an intended change over time and the perspective of the intended change (for example of the patient or of society) play an important role. Mindful of the adage “all improvement involves change, not all changes are improvement” we think improvement is the combined and unceasing efforts of everyone – e.g. healthcare professionals, patients and their families, researchers, payers, planners and educators – to make the changes that will lead to better patient outcomes, better system performance and better professional development [15].

The concept of the learning organization was first introduced by Peter Senge, who identified five disciplines that characterize learning organizations: systems thinking, personal mastery, mental models, building shared vision, team learning, and systems thinking [16]. A learning organization is skilled in both learning and realizing improvement. In our view there is a learning organization if positive changes take place in line with the five disciplines. This fosters learning and enables organisations to continually improve and adapt to succeed and thrive in a changing environment [17, 18].

Both learning and improvement have a cyclical character and mutually reinforce each other [11, 12]. Going through multiple cycles of learning and improvement means that knowledge and experiences are continuously processed creating new information. New information can lead to a better understanding of the causes and consequences of problems that arise and to the development of possible solutions. Implementing such a solution can lead to improvement. By going through this cycle over and over again, new knowledge and continuous improvement appear, but even if there is no improvement, de facto new knowledge is created.

Learning within organizations contributes to solving problems and ultimately to better performance [19–22]. Organizational learning and continuous improvement enhance each other, and by going through multiple cycles of learning and improvement, knowledge and experiences are continuously processed, creating new information. To enable organizational learning, the presence of learning capability is necessary [23, 24]. Likewise, the continuous improvement of healthcare requires improvement capability [25]. In this study we focus on the organisational attributes that contribute to L&IC. We define organizational attributes as well-considered qualities or features of a system, such as a healthcare organization.

Research into organizational attributes that contribute to L&IC seems to be based more on theory than on empirical research. An example of this is Kaplans study on the Model for Understanding Success in Quality (MUSIQ) [26] that is mainly aimed at contributing to theory development. Another potential relevant theoretical study is the presentation of the Consolidated Framework For Implementation Research (CFIR) [27] that offers an overarching typology to promote implementation theory development and verification about what works where and why across multiple contexts. Kaplan focuses on contextual factors that influence the success of quality improvement projects. Damschroder presents a framework to understand the dynamic, multi-level, transient nature of implementation of interventions in specific contexts such as clinical trials. In our study however, we focus on the healthcare organization as a whole. We seek to enrich the scholarly knowledge with this review of empirical research conducted in healthcare organizations. We do not discuss learning processes as such or the effectiveness of specific improvement methods. In doing so, we hope to provide more insight into the organizational attributes that contribute to L&IC of healthcare organizations.

Studying L&IC is relevant not only for healthcare organizations and healthcare professionals but in particular also for regulators [28]. There is growing awareness that quality of care requires more than just compliance and that, as became apparent during the COVID-19 crisis. It is precisely the ability to adapt to challenges and changes that plays an important role in maintaining the high quality of care. L&IC contribute to this adaptability. This highlights the importance of L&IC and the ability to understand which organizational attributes contribute to it. However, it currently remains unclear how to assess whether healthcare organizations are sufficiently capable of learning and improving. Moreover, there are no unambiguous and generally accepted definitions for the concepts of learning capability and improvement capability [24, 29]. For this study, we kept the following definitions in mind: learning capability refers to patterns of action that allow an organization to process knowledge and experience, generate new knowledge bases on existing knowledge and experience, and store knowledge for later use when the need arises [30, 31]. Improvement capability is the organizational capability to intentionally and systematically use improvement approaches, methods and practices to change processes and products/services to generate better performance [29].

The aim of our research is to identify internal organizational attributes that contribute to the learning and improvement capabilities of healthcare organizations and to construct a framework to assess these factors. For this, we conducted a literature review. We first explain the selection process for this review, and then we present the results of the review itself and the synthesis of the data. Finally, we propose a framework based on this synthesis.

## Methods

### Search strategy

**Fig. 1:**
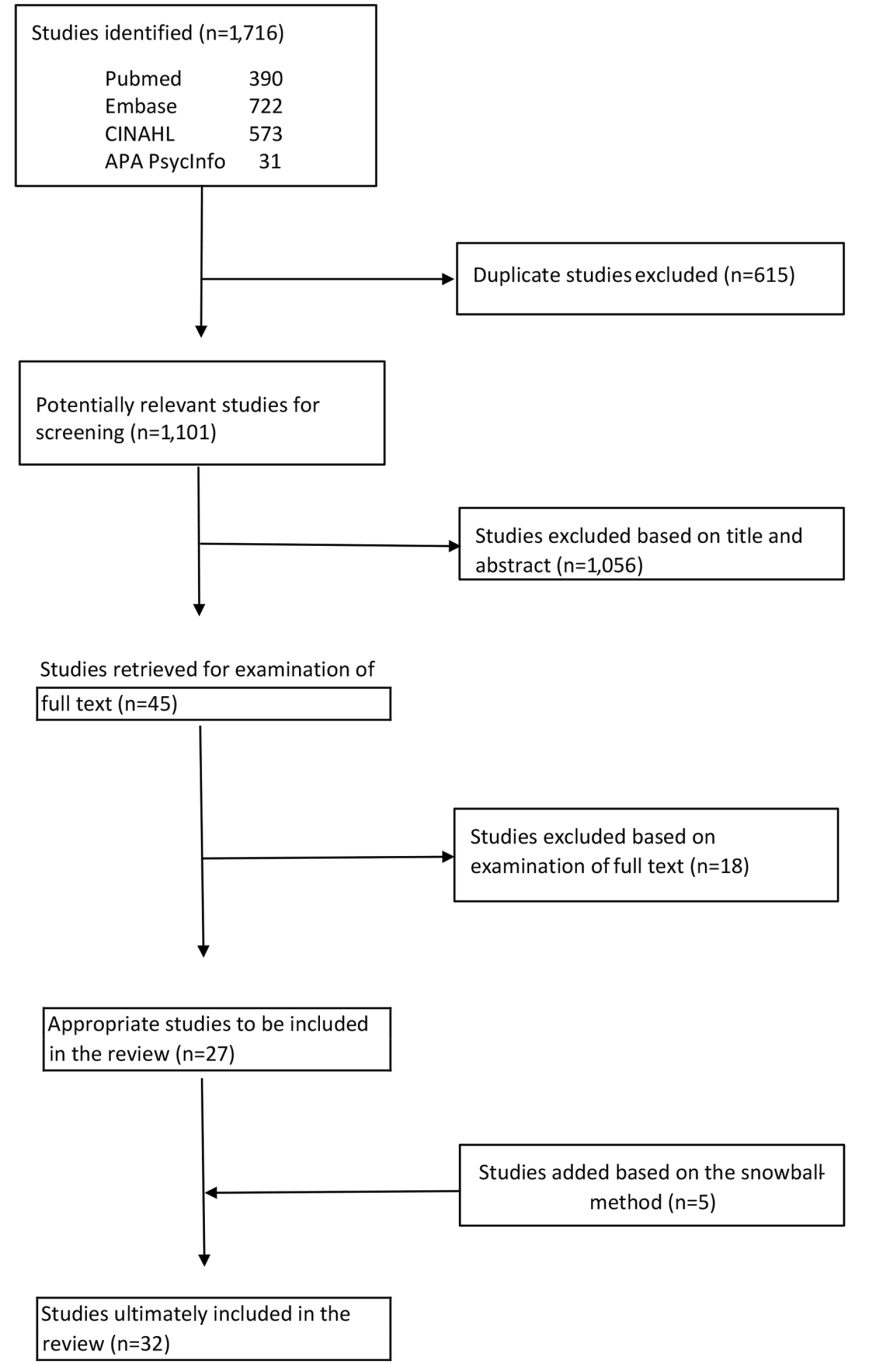
Study selection process

We developed a search strategy for PubMed, Embase, CINAHL, and APA PsycINFO. We chose three groups of keywords: setting, improvement capability and variables. We determined specific keywords for each group [see Additional file 1]. A selected article had to contain at least one keyword from each group. The search was limited to studies in English published between January 2010 and April 2020, because we wanted to have an overview of the most recent scientific knowledge in the field of LC&IC. We included eight review articles with references to articles prior to 2010 for input outside of the selected time period. On April 3, 2020, we conducted a literature search for this review [see Additional file 2]. We found 1,716 studies. After removing duplicates using Endnote, 1,101 studies remained. Figure 1 shows the complete study selection process.

### Methods of screening and selection criteria

We performed a two-step screening procedure. The first step was an initial screening of studies based on title and abstract. The screening was performed independently by two reviewers (CGdK, CK) according to two criteria: (i) the study is about one or more healthcare organizations or a healthcare inspectorate, (ii) the conclusion of the study is about improvement. Both criteria were drawn up in advance. Both reviewers assessed whether the title or abstract contained words that indicated that the article refers to both a healthcare organization and improvement. Articles about specific professional groups, for example members of the board or nurses, about specific departments, such as IC or long stay, about specific processes, like the analysis of adverse incidents, or about specific improvement methods and techniques, such as lean and six sigma, were excluded. Both reviewers could score yes/no per article and per criterium in an Excel file. If both reviewers scored yes on both criteria, the article was included. If both reviewers scored no on both criteria, the article was excluded. If there was no consensus, the researchers discussed the arguments for inclusion or exclusion in a joint consultation and reached a joint judgment.

In the first step of the screening procedure, the two reviewers achieved consensus on a vast majority of the selected articles, but there was no consensus on 70 articles. In a joint consultation, the researchers reached a joint judgment, resulting in 45 articles being included for the second step (Table 1).

**Table 1:** Results of the first screening step

	Inclusion	Exclusion	Total
Consensus	27	1004	1031
Consensus after joint consultation	18	52	70
Total	45	1056	1101

The second step of the screening procedure was to screen the full text of the articles. CGdK and CK again independently assessed the included articles using the same criteria as in the first step. Both reviewers assessed whether the research in the article is about one or more healthcare organizations and whether the conclusion relates to improvement. Both reviewers could score yes/no per article and per criterium in an Excel file. If both reviewers scored yes on both criteria, the article was included. If both reviewers scored no on both criteria, the article was excluded. If there was no consensus, the researchers discussed the arguments for inclusion or exclusion in a joint consultation and reached a joint judgment.

In this second step, the two reviewers achieved consensus on 35 articles. There was no consensus on 10 articles. In a joint consultation, the two reviewers reached a joint judgment to include four (Table 2).

**Table 2:** Results of the second screening step

	Inclusion	Exclusion	Total
Consensus	23	12	35
Consensus after joint consultation	4	6	10
Total	27	18	45

Finally, we added five studies identified through scanning of the references of the included articles (snowball method).

### Data extraction

We executed the data extraction in two steps. We carefully read each article and systematically summarized it. We then judiciously extracted the data without using a formal protocol. First we made an Excel file with two categories: (1) findings that contribute to L&IC and (2) findings that hinder L&IC. For each article, we made a detailed overview of the findings and assigned them to one of two categories. We then grouped the findings step-by-step into higher, more general-level categories using an interpretive approach until categories emerged that were sufficiently different from each other while also being internally consistent. This synthesis has been discussed by the authors.

### Patient and public involvement

Clients, as well as other groups mentioned in the literature, are not involved, as this research is purely a review of the scholarly literature.

## Results

We included 32 articles published between January 2010 and April 2020 [see Additional file 3 for full references]. The articles are based on different research methods, ranging from mixed-method designs and qualitative and quantitative studies to conceptual papers and a case study. Eight of the 32 included studies were reviews. The studies included describe the results of research in a variety of settings, such as hospitals, mental health organizations, not-for-profit healthcare organizations, the British NHS, the American Veterans Health Administration, primary care, long-term care and one regulator.

We inductively identified five organizational attributes that contribute to L&IC in healthcare organizations. Keeping in mind the lessons from Peters and Waterman [32] - to distinguish between hardware (strategy, structure and systems) and software elements of an organization (culture and shared values, (core) skills and style) - we looked per attribute for concrete behaviours or structures (Table 3). We list the five attributes in random order: perceived leadership commitment (37 findings), open culture (50 findings), room for team development (74 findings), initiating and monitoring change (98 findings) and strategic client focus (14 findings). For each organizational attribute, we found facilitators that contribute to L&IC but also some barriers that hinder L&IC. In this section, we present the five attributes and describe the main findings. Table 4 provides an overview. Based on the analysis of the five attributes, we constructed a framework that relates organizational attributes to L&IC.

**Table 3:** Overview of the included studies

AUTHOR	YEAR	COUNTRY	METHOD	ORGANIZATIONAL SETTING	ORGANIZATIONAL ATTTRIBUTES				
				(number)	**Perceived leadership commitment**	**Open culture**	**Room for team development**	**Initiating & monitoring change**	**Strategic client focus**
Alexander	2011	USA	Review (n = 107)	Health Care Organizations		Supportive attitude	Education and training Motivation Capacitiy management Behavior of team members Collaboration	Process development Feedback loop	
Babich	2016	USA	Mixed-method design	Health care organization grant recipients (29)		Supportive attitude Encouraging behavior Feedback and reflection	Education and training	Feedback loop Resources and infrastructure	
Balasubra-manian	2015	USA	Methodology	Delivery of healthcare Primary care organizations (9)		Feedback and reflection		Process development Feedback loop	Client orientation
Berta	2015	Canada	Conceptual paper			Interaction Feedback and reflection		Process development Willingness to change Feedback loop Resources and infrastructure	Service orientation
Dalmas	2019	Malta	Qualitative study	Healthcare system Hospital (1)	Supportive leadership Strategic leadership		Motivation Behavior of team members	Willingness to change Resources and infrastructure	
Doyle	2013	UK	Quantitative study	NHS frontline care teams (19)	Supportive leadership Strategic leadership		Balanced team	Process development Willingness to change Feedback loop Resources and infrastructure	Client orientation
Eljiz	2018	Australia	Conceptual paper	Healthcare			Education and training		Client orientation
Evans	2016	Canada	Mixed-method design, incl. review (n = 114)	Organizational context Organizational capabilities Integrated care Integrated delivery system Integrated care initiative	Supportive leadership	Interaction		Willingness to change Resources and infrastructure	Client orientation Service orientation
Evans	2017	Canada	Review (n = 47)	Health services management Cancer Care Ontario (1)		Interaction Encouraging behavior	Education and training	Willingness to change Feedback loop Resources and infrastructure	
Fieldston	2016	USA	Mixed-method design	Hospital (1)	Supportive leadership Strategic leadership	Encouraging behavior	Education and training Balanced team	Feedback loop Resources and infrastructure	
Foley	2017	UK USA	Mixed-method design, incl. review (n = 92)	Health system and a research facility				Process development	
Furnival	2017	UK	Review (n = 70)		Supportive leadership Strategic leadership	Interaction	Motivation	Process development Feedback loop	Client orientation
Furnival	2018	UK	Qualitative study	UK regulatory agencies (6)	Strategic leadership			Process development	
Greenfield	2010	Australia	Mixed-method design	Aged care and rehabilitation service (1)		Interaction Supportive attitude Encouraging behavior Feedback and reflection	Education and training Motivation Collaboration	Feedback loop	Client orientation Service orientation
Guzman	2015	Australia	Conceptual paper					Process development	Service orientation
Harvey	2015	UK	Qualitative study	NHS organization (3)	Strategic leadership	Interaction Encouraging behavior Feedback and reflection	Motivation	Process development Resources and infrastructure	
Hernandez	2013	USA	Mixed-method design, incl. review (n = 27)	Not-for-profit multihospital integrated delivery system (1)	Strategic leadership	Supportive attitude Feedback and reflection	Capacity management	Willingness to change Feedback loop	Client orientation
Höög	2016	Sweden	Longitudinal case study	Specialized health care provider (1)		Feedback and reflection		Process development Willingness to change	
Jeffs	2016	Canada	Qualitative study	Collaboration Communities of practice Point-of-care Acute care hospital (2) Long term care center (1) Mental health science center (1)			Education and training Behavior of team members	Feedback loop	
Kaplan	2010	USA	Review (n = 47)	Healthcare	Supportive leadership Strategic leadership		Motivation Balanced team	Resources and infrastructure	
Kilbourne	2019	USA	Conceptual paper	Veterans Health Administration		Interaction	Capacity management	Process development Willingness to change Feedback loop	
Kislov	2014	UK	Conceptual paper	Organizational capabilities Capability development	Supportive leadership Strategic leadership	Interaction Supportive attitude Feedback and reflection	Capacity management	Willingness to change	
Kringos	2015	UK	Umbrella review of systematic reviews (n = 56)		Supportive leadership Strategic leadership	Interaction Supportive attitude Encouraging behavior	Education and training Motivation Capacity management Balanced team	Process development Willingness to change Resources and infrastructure	
Lanteigne	2016	Anguilla Italy	Mixed-method design	Health Authority and a hospital (2)	Supportive leadership Strategic leadership	Feedback and reflection	Education and training Capacity management Behavior of team members Collaboration	Willingness to change Feedback loop Resources and infrastructure	
Leufvén	2015	Nepal	Quantitative study	Health systems Hospital (1)		Feedback and reflection	Education and training Motivation	Resources and infrastructure	
Luxford	2011	USA	Qualitative study	Health Care Organizations (8)	Supportive leadership	Supportive attitude Encouraging behavior Feedback and reflection	Motivation Capacity management	Process development Feedback loop Resources and infrastructure	Client orientation
Potts	2017	USA	Mixed-method design	Integrated healthcare (1)	Strategic leadership	Encouraging behavior	Balanced team	Willingness to change	
Psek	2015	USA	Conceptual paper	Delivery system Health/wellness organization (1)	Strategic leadership	Interaction Feedback and reflection		Feedback loop Resources and infrastructure	Client orientation
Schilling	2010	USA Sweden	Quantitative study	Sites of not-for-profit health organizations (n = 5)	Strategic leadership	Supportive attitude		Willingness to change	
Schilling	2011	USA	Mixed-method design	Not-for-profit health plan (1)	Supportive leadership		Education and training	Feedback loop	
Shea	2018	USA	Qualitative study	Primary care Healthcare teams Organizational innovation Primary care practices (10)	Supportive leadership	Supportive attitude	Capacity management Collaboration	Willingness to change	
Singer	2012	USA	Quantitative study	Veteran Health Admin. (1) Mental health organizations (2)	Supportive leadership	Supportive attitude Feedback and reflection	Education and training	Process development Feedback loop	

**Table 4:** Summary of organizational attributes of L&IC and their facilitators and barriers

ORGANIZATIONAL ATTRIBUTES	FACILITATORS	BARRIERS
**Perceived leadership commitment**	Supportive leadership	Hierarchical leadership
	Strategic leadership	High turnover of leaders
		Lack of alignment of priorities
**Open culture**	Encouraging behavior	Lack of facilities for reflection
	Supportive attitude	Difficult communication
	Feedback and reflection	
	Interaction	
**Room for team development**	Education and training	Excessive workload
	Motivation	High turnover of staff
	Capacity management	Excessive deployment of external employees
	Behavior of team members	
	Balanced team	
	Collaboration	
**Initiating & monitoring change**	Process development	Inability to finalize plans
	Willingness to change	Lengthy transitions
	Feedback loop	
	Resources & infrastructure	
**Strategic client focus**	Client orientation	Difficulties in changing employee mindsets
	Service orientation	Long time needed for change

### Organizational attributes that contribute to L&IC in healthcare organizations

#### Perceived leadership commitment

We identified two facilitators of perceived leadership commitment: supportive leadership and strategic leadership. Several researchers use not only the word commitment but also engagement, (visible) support, recognition and listening [33–37]. The reviews conducted by Kaplan [38] and Alexander [39] show that both commitment of board leadership and commitment of team leadership have consistent associations with improvement capability. Luxford [33] seems to confirm this, as do Fieldston [40] and Shea, [41] who identified strong committed support of the board and senior staff as organizational attributes that contribute to improvement. To achieve long-term successful improvement, not only is the continued commitment of the board and senior staff critical [42] but also strategic leadership behaviors, such as providing direction, developing a new vision and inspiring people [36, 43–45]. A number of studies also mention leadership behavior that hinders the development of L&IC. Leufvén [46] found centralized, hierarchical leadership a barrier, and Fieldston [40] identified frequent changes in senior leaders having a negative influence on L&IC. In a qualitative survey of 24 healthcare employees, both physicians and nonphysicians, from 10 primary care practices, Shea [41] revealed a lack of alignment of strategic and operational priorities by the leadership and staff as a barrier.

#### Open culture

We found clear indications that an open organizational culture positively affects an organization’s ability to learn, innovate, diffuse, and sustain quality improvement [29, 38, 47]. We identified four facilitators of an open culture: encouraging behavior, a supportive attitude, feedback and reflection, and interaction. The first facilitator we found is encouraging behavior, such as tolerance for ambiguity and unconventionality, including different perspectives in complex challenges and the creation of a patient safety culture by stimulating open discussions about mistakes [35, 40, 45, 48, 49]. A second facilitator that we found is a supportive attitude of employees towards change, learning and improvement [33, 37, 47, 50]. This underlines our previous finding that not only supportive leadership but also employee engagement is important. A third facilitator of an open culture is feedback and reflection. This means there is room for dialogue and inquiry, encouragement of self-assessments and teams taking time for reflection over a long period of time [23, 36, 37, 43, 46–49, 51]. We found interaction to be a fourth facilitator of an open culture. Interaction stands for a collaborative attitude towards people inside or outside the organization and towards other organizations. It also refers to building and maintaining partnerships and networks and involving stakeholders [23, 29, 43, 44, 50, 52]. Some studies have identified barriers to an open culture that hinder the development of L&IC. Kislov [50] mentioned a lack of reflection that can limit responsiveness to the ever-changing context. Barriers mentioned by Höög [53] are a lack of facilities for reflection and difficulties in finding efficient channels for communication between the many organizational levels.

### Room for team development

We identified six facilitators of room for team development: motivation, education and training, capacity management, behavior of team members, a balanced team, and collaboration. The first facilitator we found is staff motivation. This implies willingness to learn and change [33, 38, 43, 54]. The second facilitator, education and training, refers mainly to common learning. According to Leufvén [46] team learning is a basic facilitator of a learning organization. Team members learn together. They share less formalized knowledge and ideas and expose each other to new concepts [36, 48, 55, 56]. We found capacity building to be a third facilitator. This facilitator is about capacity management, workload, focus on employee satisfaction, and investment in resources such as protected time for staff and appropriate reward systems [33, 35, 39, 50]. The fourth facilitator concerns the behavior of team members and relates to acquiring and practicing leadership skills, improving autonomy and directly applying new knowledge in practice [36, 56]. A balanced team is the fifth facilitator and mainly involves multidisciplinary composition of teams and to value the wisdom of a group of individuals over that of one expert [35, 45]. The final facilitator of room for team development is collaboration. This facilitator relates to the organizational climate for teamwork, the attitude of employees towards teamwork and the extent to which the organization promotes teamwork. Collaboration also has to do with team cohesion and the way professionals communicate with each other and in teams [41]. We found excessive workload, high staff turnover and excessive use of external staff members as barriers to room for team development [35, 39].

### Initiating and monitoring change

We identified four facilitators of initiating and monitoring change: willingness to change, process development, feedback loop, and resources & infrastructure. We found some features of willingness to change that are positively related to initiating and monitoring change: a strong orientation towards innovation and improvement and the willingness to develop new services [50, 52, 57]. Other features include understanding the limitations of planned change and seizing opportunities through unexpected changes [35, 45, 58]. A second facilitator is process development. Healthcare organizations have to adapt to ever-changing circumstances and, therefore, have a continuous need to develop their quality of care and business processes [43, 58]. For this reason, organizations that facilitate activities to design better processes by experimentation, conducting pilots, developing and adapting improved processes are associated with greater L&IC [23, 34, 37]. The third facilitator is the presence of feedback loops in healthcare organizations. This refers to using systematic methods to manage change and to make improvements through constant experimentation and reflection [23, 29, 59]. The plan-do-study-act cycle enables a systematic and continuous feedback loop and includes a data collection and reporting process to monitor implementation progress and organizational performance [23, 37, 48, 49, 58, 60]. It is worth noting that data collection is not limited to quantitative data but also includes qualitative data, such as exchanging stories about what worked well or what did not [56]. The fourth facilitator is resources and infrastructure. Several studies highlight the importance of sufficient resources such as funding, administrative support, and the availability, functionality and use of information technology systems [35, 38, 43, 46, 52]. Babich [47] and Evans [52] also emphasize the relevance of formal systems and processes such as codified knowledge, experience and routines stored in databases and written documentation, e.g., policies, procedures, and protocols. We also found impeding attributes for initiating and monitoring change, such as the inability to finalize plans and lengthy transitions [33, 54].

### Strategic client focus

We identified two facilitators of strategic client focus: client orientation and service orientation. Client orientation is about the importance of client-centeredness and the involvement of clients and family in defining what quality of care should look like. Luxford [33], in their study of eight healthcare organizations with a reputation for successfully promoting client-centered care, found that organizations that succeeded in advancing client-centered care had made client focus an explicit part of both the strategic vision and leadership communication. Leadership clearly and consistently communicated the importance of client-centeredness to every member of the organization. Hernandez [49] also mentioned a clear and internally consistent organizational mission and an aligned organizational strategy as organizational features that contribute to initiating client-centered innovation. Greenfield [48] and Eljiz [61] found client-centered care related to improvement, and Psek [44] stated in his conceptual paper that client and family engagement in the learning process is critical to achieving better outcomes. The other facilitator of strategic client focus is service orientation. This means that a strategic focus on manageable and reproducible processes encourages maximizing service and enables best practices and service delivery [23, 57, 62]. Luxford [33] highlighted some barriers to strategic client focus, such as difficulties in changing employee mindsets from organizational focus to client focus and the time needed for that change, given that cultural change does not happen quickly.

### Framework

**Fig. 2:**
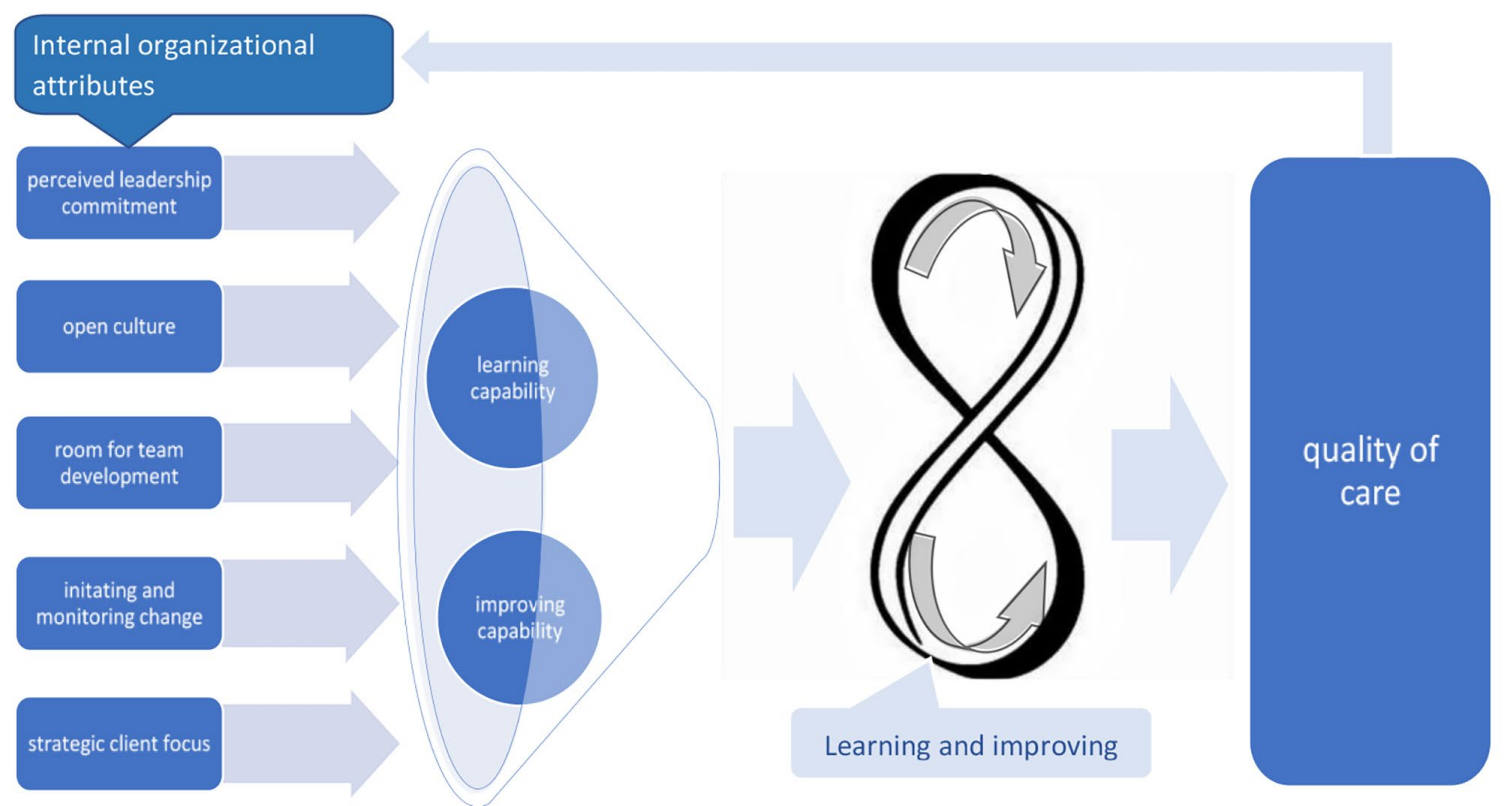
Framework for Learning & Improvement Capabilities

Based on the analysis of the five identified attributes, we constructed a framework (Fig. 2) that relates organizational attributes to the learning capability and the improvement capability of healthcare organizations. The framework helps to understand that these five attributes influence the learning and improvement process of healthcare organizations the quality of the care they provide and the feedback loop that exists between them. The framework can provide guidance for the assessment of L&IC of healthcare organizations.

## Discussion

The aim of our scoping review was to identify internal organizational attributes that contribute to the L&IC of healthcare organizations. We identified five: perceived leadership commitment, open culture, room for team development, initiating and monitoring change, and strategic client focus. Each attribute consists of multiple facilitating aspects. We also found some hindering aspects.

In our search we explicitly focussed on internal organizational attributes. This presumes a sharp distinction between what Damschroder calls ‘inner and outer setting’. The CIFR shows that no sharp boundary can be drawn. The findings in our study seem to confirm this. An example is interaction as a facilitator of an open culture. Interaction does not only take place within an organization, but also outside, for example when building partnerships across organisational boundaries. Nonetheless we feel this fits with inner setting, because culture is really an organizational attribute.

We have not looked at the learning or improvement processes of care providers or what specific contribution certain professional groups make to this. We also did not look at the effectiveness of methodologies or interventions. Much has already been published on these topics [63–70]. The significance of the five attributes we discovered is that they do not solely pertain to a single department, profession, or intervention, but rather provide a valuable opportunity for managing an organisation, driving change, or facilitating external regulation.

It has proven difficult to specifically define the concepts ‘learning’ and ‘improvement’, as neither have an unambiguous and generally accepted definition [29–31]. Both concepts are intertwined: organizational learning and improvement reinforce each other in a continuous and interactive process. New knowledge can inspire activities to improve care and deliver better performance, but it is not a given that new knowledge always leads to the appropriate action. For example, knowing about a new guideline does not always lead to implementing this guideline. Improvement activities do not always lead to knowledge if, for example, there is no feedback or reflection. We therefore propose that the five organizational attributes work best when they are focussed on both learning and improvement and their mutual interaction.

We have found some similarities and differences between our findings and the MUSIQ and CIFR models. The themes leadership, culture and change can also be found in MUSIQ and CIFR although the definitions of these concepts and the ordering differs. A striking difference concerns the involvement of clients or customers. Kaplan’s MUSIQ model lacks consideration for clients or customers, while the CFIR model acknowledges patient needs but not patient involvement. Our findings suggest that explicit client focus positively contributes to improvement, consistent with Psek’s assertion that patient and family engagement in learning is crucial for better outcomes [44].

Another reflection is that the presence or absence of a particular attribute in an organization is usually not absolute. An attribute can be present in one part of an organization but absent in another. Senior management, for example, can proclaim a strategic client focus, while client focus can vary between the different departments or locations of that organisation. This gap between formal structures and actual practice is referred to as decoupling [71, 72]. Furthermore, L&IC appears not only to be the sum of individual learning and improvement skills, but we learn that commitment, involvement and supportive leadership at both the team and board levels are needed. For the five attributes to have an effect on the organisation as a whole, we found they should be aligned throughout all levels of the organisation.

The main reason for this study was the lack of clarity in assessing whether healthcare organizations have sufficient L&IC. While the five attributes are formulated in general terms, the aspects underlying the attributes are remarkably concrete. We therefore believe they can be useful not only for managers to understand which aspects they have to focus on to improve L&IC but also for boards and external regulators to understand why L&IC are not equally present in all parts of an organization.

As mentioned before, we kept the distinction between hardware and software elements of an organization [32] in mind while reviewing the included studies. We note that most of the facilitators identified among the five attributes consist of software elements (e.g., supportive attitude, motivation and willingness to change); only a few are hardware elements (e.g., capacity management, resources and infrastructure). In our opinion, a possible explanation for the emphasis on software elements rather than on hardware elements is the fact that learning and improvement can be seen as psychological and social processes. Hardware elements on the other hand enable and facilitate change, but cannot initiate it. In regard to understanding or assessing L&IC, careful consideration of the appropriate method is required. Qualitative methods based on observations and stories may be more suitable for understanding L&IC, while quantitative approaches based on ‘hard data’ appear to be more useful for monitoring development. However, we think that a combination of both methods provides the most insight.

Finally, our results suggest that an aligned organizational strategy to achieve client-centeredness, family involvement and service maximization contributes to L&IC. These results are in line with recent literature [1, 7–9] that emphasizes the role of the client in the learning and improvement process of healthcare organizations.

This scoping review provides insight into organizational attributes that contribute to L&IC. A strength of this research is the methodologically sound and therefore traceable review and the selection procedure, which two researchers conducted independently of each other. Another strength is the variety of institutions reported in the included articles, making the results relevant for multiple healthcare institutions. A limitation of our study is that no critical appraisal of the included articles was performed. The result is a collection of articles with rich and heterogeneous reporting of findings. Another limitation of this study is that although two theoretical models were used to reflect on the findings, no model was used to structure the analysis of the included articles.

## Conclusion

We identified five organizational attributes that contribute to L&IC: perceived leadership commitment, open culture, room for team development, initiating and monitoring change, and strategic client focus. It would be fruitful to develop an instrument to assess the variations in each of the five attributes and their mutual interaction to be able to interpret the differences in L&IC of healthcare organizations. Future research could focus on how to adequately monitor and assess these attributes in relation to existing theoretical frameworks, as we realise this can be challenging. The use of qualitative methods seems most appropriate to understand or assess these organizational attributes and their relationship to quality of care. Research into the underexposed theme of barriers can also be useful to enrich our knowledge about organizational attributes that facilitate or hinder the development of these competencies. We feel it is also important to look more closely at how clients of healthcare organisations are involved in the development and assessment of L&IC because they are the ones that ultimately bear the brunt or reap the benefit of changes in quality of care.

## Electronic supplementary material

Below is the link to the electronic supplementary material.

Additional File 1

Additional File 2

Additional File 3

Additional File 4

## Data Availability

All data generated or analysed during this study are included in this published article and its supplementary information files [see Additional file 4].
