# Supplementary material for: Organizational attributes that contribute to the learning & improvement capabilities of healthcare organizations: a scoping review
Source: BMC Health Serv Res. 2023 Jun 7;23:585. doi: 10.1186/s12913-023-09562-w (PMC10244857; doi:10.1186/s12913-023-09562-w)
Supplement: Supplementary file 1 — Additional File 1 [file 12913_2023_9562_MOESM1_ESM.docx]

# APPENDIX 1 Specific keywords per group

| **#1** **Setting** | |
| --- | --- |
| Mesh | Health Facilities |
| tiab | healthcare provider care provider healthcare facilit* health facilit*  care facilit*  healthcare organization* healthcare setting |
| **#2** **Improvement capability** | |
| Mesh | Quality Improvement |
| tiab | improv* change transform* learning capacit* capabil* |
| **#3** **Variables** | |
| Mesh | Organizational Culture Clinical Governance  Safety Management  Change Management  Total Quality Management |
| tiab | contextual factor* barrier*/facilitat*/enabler* corporate culture*  organizational climate/learning/factor governance  learning culture/health system*/health care system* knowledge transfer* innovation/change management leadership*  managerial commitment/skill*  process improv*/learning/performance*/implementation/management performance improvement quality/safety/error management  safety culture* hazard management  customer/client/patient/user focus/involv*  stakeholder/supplier/partnership participat*/collaboration/involv* |
