# Supplementary figures and images for: Organizational attributes that contribute to the learning & improvement capabilities of healthcare organizations: a scoping review
Source: BMC Health Serv Res. 2023 Jun 7;23:585. doi: 10.1186/s12913-023-09562-w (PMC10244857; doi:10.1186/s12913-023-09562-w)

# APPENDIX 2 Full electronic search strategy for the PubMed database


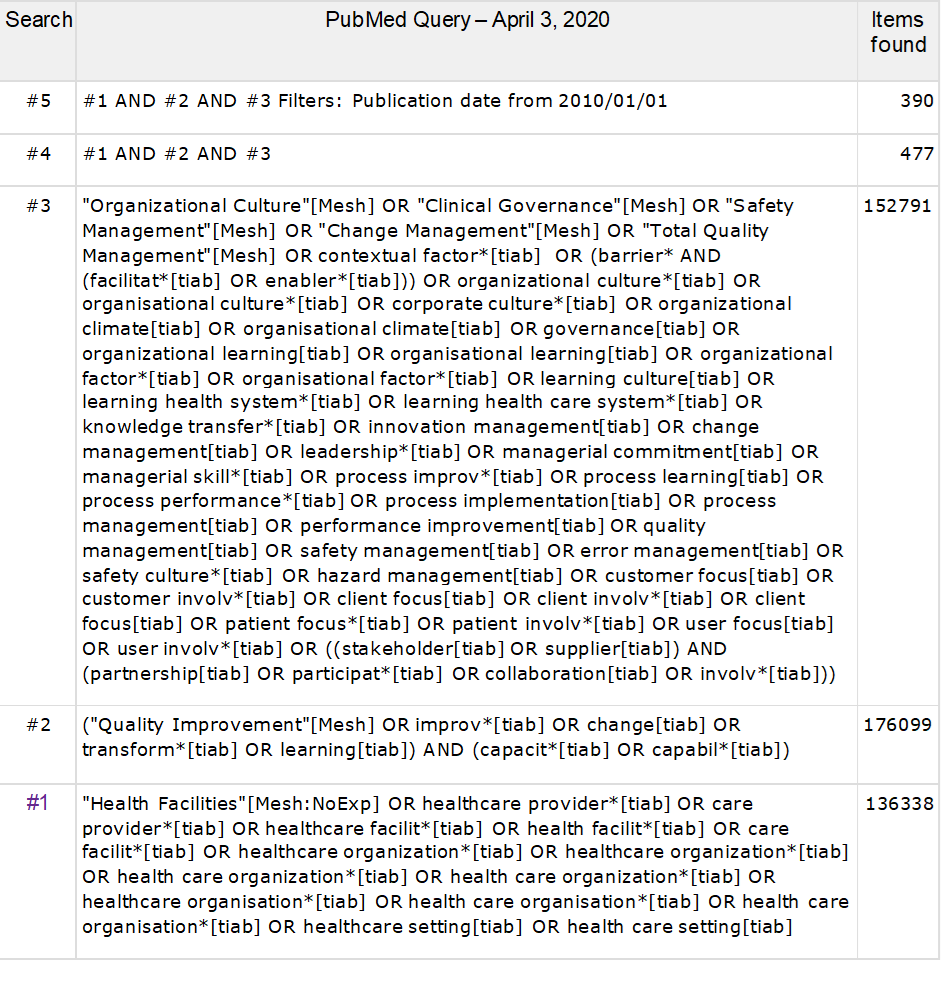

Supplement: Supplementary file 2 — Additional File 2 [file 12913_2023_9562_MOESM2_ESM.docx]
