# Supplementary material for: Organizational attributes that contribute to the learning & improvement capabilities of healthcare organizations: a scoping review
Source: BMC Health Serv Res. 2023 Jun 7;23:585. doi: 10.1186/s12913-023-09562-w (PMC10244857; doi:10.1186/s12913-023-09562-w)
Supplement: Supplementary file 3 — Additional File 3 [file 12913_2023_9562_MOESM3_ESM.docx]

# APPENDIX 3 Full references of articles in review (n=32)

Alexander JA, Hearld, LR. The science of quality improvement implementation: developing capacity to make a difference. *Med Care* 2011; S6-S20. <https://www.jstor.org/stable/23053716>

Babich LP, Charns MP, McIntosh N, *et al*. Building systemwide improvement capability: Does an organization's strategy for quality improvement matter? *Qual Manag Health Care* 2016**;25**(2):92-101. <https://doi.org/10.1097/QMH.0000000000000089>

Balasubramanian BA, Cohen DJ, Davis MM, *et al.* Learning Evaluation: blending quality improvement and implementation research methods to study healthcare innovations. *Implement Sci* 2015;**10:**31. <https://doi.org/10.1186/s13012-015-0219-z>

Berta W, Cranley L, Dearing JW, *et al*. Why (we think) facilitation works: insights from organizational learning theory. *Implement Sci* 2015;**10**:141. <https://doi.org/10.1186/s13012-015-0323-0>

Dalmas M, Azzopardi JG. Learning from experience in a National Healthcare System: Organizational dynamics that enable or inhibit change processes. *International Journal for Quality in Health Care*. 2019;**31**(6):426-432. <https://doi.org/10.1093/intqhc/mzy204>

Doyle C, Howe C, Woodcock T, *et al.* Making change last: applying the NHS institute for innovation and improvement sustainability model to healthcare improvement. *Implement Sci* 2013;**8:**127. <https://doi.org/10.1186/1748-5908-8-127>

Eljiz K, Greenfield D, Molineux J, *et al.* How to improve healthcare? Identify, nurture and embed individuals and teams with "deep smarts" *J Health Organ Manag* 2018;**32**(1):135-143.

<https://doi.org/10.1108/JHOM-09-2017-0244>

Evans JM, Brown A, Baker GR. Organizational knowledge and capabilities in healthcare:

Deconstructing and integrating diverse perspectives. *SAGE Open Med.* 2017;**5.** <https://doi.org/10.1177%2F2050312117712655>

Evans JM, Grudniewicz A, Baker GR, *et al.* Organizational context and capabilities for integrating care: A framework for improvement. *Int J Integr Care.* 2016;16(3). <https://doi.org/10.5334%2Fijic.2416>

Fieldston ES, Jonas JA, Lederman VA, *et al.* Developing the capacity for rapid-cycle improvement at a large freestanding children's hospital. *Hospital Pediatrics* 2016;**6**(8):441-448. <https://doi.org/10.1542/hpeds.2015-0239>

Foley TJ, Vale L. What role for learning health systems in quality improvement within healthcare providers? *Learn Health Syst.* 2017;**1**(4):e10025. <https://doi.org/10.1002/lrh2.10025>

Furnival J, Walshe K, Boaden R. Conceptualising and assessing improvement capability: a review. *Int J*

## *Qual Health Care* 2017; 29:604–11. <https://doi.org/10.1093/intqhc/mzx088>

Furnival J, Boaden R, Walshe K. Assessing improvement capability in healthcare organisations: a qualitative study of healthcare regulatory agencies in the UK. *Int J Qual Health Care* 2018;**30**(9):715723. <https://doi.org/10.1093/intqhc/mzy085>

Greenfield D, Nugus P, Travaglia J. Auditing an organization's interprofessional learning and interprofessional practice: the interprofessional praxis audit framework (IPAF). *Journal of Interprofessional Care* 2010;**24**(4):436-449. <https://doi.org/10.3109/13561820903163801>Guzman G, Fitzgerald JA, Fulop L, *et al.* How best practices are copied, transferred, or translated between health care facilities: A conceptual framework. *Health Care Manage Rev* 2015;**40**(3):193202. <https://www.jstor.org/stable/48516407>

Harvey G, Jas P, Walshe K. Analysing organisational context: Case studies on the contribution of absorptive capacity theory to understanding inter-organisational variation in performance improvement. *BMJ Quality and Safety* 2015;**24**(1):48-55 [http://dx.doi.org/10.1136/bmjqs-2014002928](http://dx.doi.org/10.1136/bmjqs-2014-002928)

Hernandez SE, Conrad DA, Marcus-Smith MS, *et al.* Patient-centered innovation in health care organizations: a conceptual framework and case study application. *Health Care Manage Rev* 2013;**38**(2):166-175. <https://www.jstor.org/stable/48516155>

Höög E, Lysholm J, Garvare R, *et al.* Quality improvement in large healthcare organizations. *J Health Organ Manag* 2016;**30**(1)133-153. <https://doi.org/10.1108/JHOM-10-2013-0209>

Jeffs L, McShane J, Flintoft V, *et al.* Contextualizing learning to improve care using collaborative communities of practices. *BMC Health Services Research* 2016;**16**:464. <https://doi.org/10.1186/s12913-016-1566-4>

Kaplan HC, Brady PW, Dritz MC, et al. The influence of context on quality improvement success in health care: a systematic review of the literature. The Milbank Quarterly 2010,88(4):500-59. <https://doi.org/10.1111/j.1468-0009.2010.00611.x>

Kilbourne AM, Goodrich DE, Miake-Lye I, *et al.* Quality enhancement research initiative implementation roadmap: Toward sustainability of evidence-based practices in a Learning Health System. *Med Care* 2019;**57:**S286-S293. <https://doi.org/10.1097%2FMLR.0000000000001144>

Kislov R, Waterman H, Harvey G, *et al.* Rethinking capacity building for knowledge mobilisation: developing multilevel capabilities in healthcare organisations. *Implement Sci* 2014;**9**:166. <https://doi.org/10.1186/s13012-014-0166-0>

Kringos DS, Sunol R, Wagner C, *et al.* The influence of context on the effectiveness of hospital quality improvement strategies: a review of systematic reviews. *BMC health services research* 2015;**15**:277. <https://doi.org/10.1186/s12913-015-0906-0>

Lanteigne G, Bouchard C. Is the introduction of an accreditation program likely to generate organization-wide quality, change and learning? *The International Journal of Health Planning and Management* 2016;**31**(3): e175-e191. <https://doi.org/10.1002/hpm.2314>

Leufvén M, Vitrakoti R, Bergstrom A, *et al.* Dimensions of Learning Organizations Questionnaire (DLOQ) in a low-resource health care setting in Nepal. *Health Res Policy Syst* 2015;**13**(6). <https://doi.org/10.1186/1478-4505-13-6>

Luxford K, Safran DG, Delbanco T. Promoting patient-centered care: a qualitative study of facilitators and barriers in healthcare organizations with a reputation for improving the patient experience*. International Journal for Quality in Health Care* 2011;**23**(5):510-515. <https://doi.org/10.1093/intqhc/mzr024>

Potts J, Thompson R, Merchant R, *et al.* Learning: Contemplating the unexamined core of Learning Health Systems. *Learn Health Syst* 2017;**1(**4):e10036. <https://doi.org/10.1002/lrh2.10036>

Psek WA, Stametz RA, Bailey-Davis LD, *et al.* Operationalizing the Learning Health Care System in an integrated delivery system. *Egems* 2015;**3**(1). <https://doi.org/10.13063%2F2327-9214.1122>

Schilling L, Chase A, Kehrli S, *et al.* Kaiser Permanente’s performance improvement system, part 1: from benchmarking to executing on strategic priorities. *The Joint Commission Journal on Quality and Patient Safety* 2010;**36**(11);484-AP5. <https://doi.org/10.1016/S1553-7250(10)36072-7>

Schilling L, Dearing JW, Staley P, *et al.* Kaiser Permanente’s performance improvement system, part

4: creating a learning organization. *The Joint Commission Journal on Quality and Patient Safety*

## 2011;**37**(12):532-AP5. <https://doi.org/10.1016/S1553-7250(11)37069-9>

Shea CM, Turner K, Albritton J, *et al.* Contextual factors that influence quality improvement implementation in primary care: The role of organizations, teams, and individuals. *Health Care Manage Rev* 2018;43(3):261-269. <https://doi.org/10.1097%2FHMR.0000000000000194>

Singer SJ, Moore SC, Meterko M, *et al.* Development of a short-form Learning Organization Survey: the LOS-27. *Med Care Res Rev* 2012;**69**(4):432-459. <https://doi.org/10.1177%2F1077558712448135>
